# Parasitic infections and medical expenses according to Health Insurance Review Assessment claims data in South Korea, 2011–2018

**DOI:** 10.1371/journal.pone.0225508

**Published:** 2019-11-26

**Authors:** Ju Yeong Kim, Myung-hee Yi, Tai-Soon Yong

**Affiliations:** 1 Department of Environmental Medical Biology, Institute of Tropical Medicine, Arthropods of Medical Importance Resource Bank, Yonsei University College of Medicine, Seoul, Korea; 2 Brain Korea 21 PLUS Project for Medical Science, Yonsei University College of Medicine, Seoul, Korea; Universidade do Estado do Rio de Janeiro, BRAZIL

## Abstract

**Introduction:**

In South Korea, Health Insurance Review and Assessment claims data contain comprehensive information on healthcare services for almost the entire population. The present study used claims data on parasitic diseases from 2011 to 2018, and associated medical expenses to investigate infection trends associated with endemic parasitic diseases in South Korea, including those not monitored by Korea Centers for Disease Control and Prevention.

**Methods:**

Basic data regarding each parasitic disease were curated from the Healthcare Bigdata Hub (http://opendata.hira.or.kr). Ten endemic parasitic diseases, three pandemic protozoan diseases, and three ectoparasitic diseases were evaluated between 2011 and 2018. Data on each parasitic disease included the number of patients of each sex, age range within 5 years, province, and total medical expenses. Heatmap and principal component analysis were performed to visualize the incidence pattern of parasitic diseases by provinces.

**Results:**

Clonorchiasis and pinworm infections decreased remarkably from 6,097 and 4,018 infections in 2011 to 3,008 and 1,988 infections in 2018, respectively. Other endemic parasitic diseases mostly declined or remained steady over the 8-year period, except for anisakiasis, which doubled from 409 in 2011 to 818 in 2018. Provinces close to North Korea had a higher frequency of claims for *Plasmodium vivax* infection. The highest rate of clonorchiasis was in Gyeongsangnam-do, while that of anisakiasis was in southern Korea. Jeju province had the highest number of claims for cysticercosis, anisakiasis, pinworm infection, and soil-transmitted helminth infections. The total medical expense for anisakiasis was 65 million Korean won (57,000 US$) in 2011, rising to 237 million Korean won (206,000 US$) in 2018. The medical expense for trichomoniasis was 6,063 million won and for scabies was 1,669 million won in 2018. Since the claims data include only data reported by healthcare providers, some discrepancies might have occurred.

**Conclusion:**

Our findings provide the basis for a health policy to reduce further infections and medical expense.

## Introduction

A variety of parasitic diseases continue to cause harm and impose medical expenses in South Korea. By the late 1960s, soil-transmitted helminths (STHs) such as *Ascaris lumbricoides*, *Trichuris trichiura*, and hookworm were highly prevalent in South Korea. Most STHs have now declined and are well controlled [1,2]. Pinworm (*Enterobius vermicularis*) infections continue to occur, usually in children in the lower grades of elementary school and preschool. The prevalence of the liver fluke (*Clonorchis sinensis*) remains around 1.4%; this parasite is transmitted through the consumption of freshwater fish and is the most prevalent helminthic parasite in South Korea [3]. In addition, there are many cases of *Anisakis* spp. infections, transmitted by eating raw sea fish [4]. *Plasmodium vivax* infections have re-emerged since 1993 and it has become one of the most important parasitic diseases in South Korea [5].

Korea Centers for Disease Control and Prevention (KCDC) has designated these diseases as National Notifiable Infectious Diseases. Malaria also falls within the mandatory surveillance system and any cases diagnosed by medical institutions should be reported to the government. *A*. *lumbricoides*, *T*. *trichiura*, pinworm, *C*. *sinensis*, and *Paragonimus* spp. infections fall under the sentinel surveillance system, monitored by designated medical institutions. However, other parasitic diseases that attract less attention, such as anisakiasis, sparganosis, and cysticercosis, have not been monitored. In addition, protozoan infections such as trichomoniasis and ectoparasite infestations such as those caused by *Pediculus capitis*, *Phthirus pubis*, and scabies have not been monitored. There is little information on nationwide parasitic diseases that are not monitored by KCDC, except for some case reports. This information, in addition to the total medical expense due to certain parasitic diseases, can reveal the national burden of such diseases and provide justification to establish the proper policy for handling the diseases. Some parasitic infections may affect a large number of people but impose less financial burden, and vice versa.

All South Korean citizens must join the National Health Security System by law. All data about medical expenses for the medical claims collected in the process of reimbursing healthcare providers are stored and managed by the Health Insurance Review and Assessment (HIRA). HIRA contains comprehensive information pertaining to healthcare services for almost the entire South Korean population (approximately 50 million people) [6]. These data help to progress medical research [7–10].

In the present study, we used HIRA claims data to investigate infection trends and medical expenses associated with endemic parasitic diseases in South Korea, including diseases not monitored by KCDC, from 2011 to 2018, and compared this information with diseases caused by certain protozoa and ectoparasites.

## Methods

### Data source

Data were sourced from HIRA claims. South Korea has a universal health coverage system, the National Healthcare Insurance (NHI) program, which covers almost 100% of the South Korean population [11]. Medical institutions submit health care utilization information in electronic format for reimbursement purposes, and this information is integrated into the HIRA claims database. The database contains information on all patients, including demographic characteristics, ambulatory care history, principal diagnosis, and comorbidities, using the International Classification of Diseases, 10th revision (ICD-10), as well as prescriptions and procedures [10]. Access to HIRA data (http://opendata.hira.or.kr) is regulated by the Rules for Data Exploration and Utilization of the HIRA, and we used data with the approval of the HIRA data access committee. All data were delivered anonymously, and no researchers had access to any potentially identifying personal information, including names, addresses, and date of birth. We used only information designated as ‘Creative Commons Attribution Share Alike (CC BY-SA)’ from the HIRA data and all the figures and maps in manuscript were created by the authors.

Disease codes used were A06 (amoebiasis), A59 (trichomoniasis), B51 (*P*. *vivax* infection), B58 (toxoplasmosis), B69 (cysticercosis), B76 (hookworm infection), B77 (ascariasis), B79 (trichuriasis), B80 (pinworm infection), B661 (clonorchiasis), B664 (paragonimiasis), B701 (sparganosis), B810 (anisakiasis), B850 (*P*. *capitis* infestation), B853 (*P*. *pubis* infestation), and B86 (scabies). STHs included three parasites: *A*. *lumbricoides*, *T*. *trichiura*, and hookworm. Basic data on each parasitic disease were collected from the website ‘Healthcare Bigdata Hub’ (http://opendata.hira.or.kr/op/opc/olap3thDsInfo.do). This database includes claims data on diseases starting in 2010, but the present study covered years 2011 to 2018 because the data from the initial year (2010) were not considered reliable. Data on each parasitic disease included the number of patients of each gender, age range within 5 years, province, and total medical expense, which is the sum of co-payment (paid by the patient at medical institution) and medical care benefits (paid by NHI) (S1–S4 Tables).

### Statistical analysis

To determine the incidence of parasitic diseases in each province, the number of patients in each province was divided by the total population of that province. We used the 8-year median value for disease specific claims frequency in each province to generate a heatmap and for principal component analysis (PCA). The data were weighted to assign equal importance to each type of parasitic infection. The sum of the proportions of disease specific claims frequency in each of the 16 provinces was 1. Heatmap analysis and PCA were performed using R 3.5.3 with the Vegan package 2.5–4. The outline of the map of South Korea was manually drawn, and the map was colored using Microsoft Paint according to the relevant data. In the formal map, provinces with similar parasite infection patterns were painted in the same color based on the results of the heatmap and PCA. The color grades in the other map were determined by the frequency of anisakiasis in 2018.

Sejong city, newly founded in 2012, was excluded from the analysis of claims frequency by provinces because the data were not considered reliable. Its population fluctuated and was relatively small (113,117 people in 2012 and 314,126 people in 2018, http://kosis.kr).

### Ethical statement

Ethical approval was not sought for the study. All data analysed were anonymised. This study contains only the reprocessed information from HIRA open data designated ‘Creative Commons Attribution Share Alike (CC BY-SA).’

## Results

### Endemic parasitic infections in South Korea, 2011–2018

**Fig 1:**
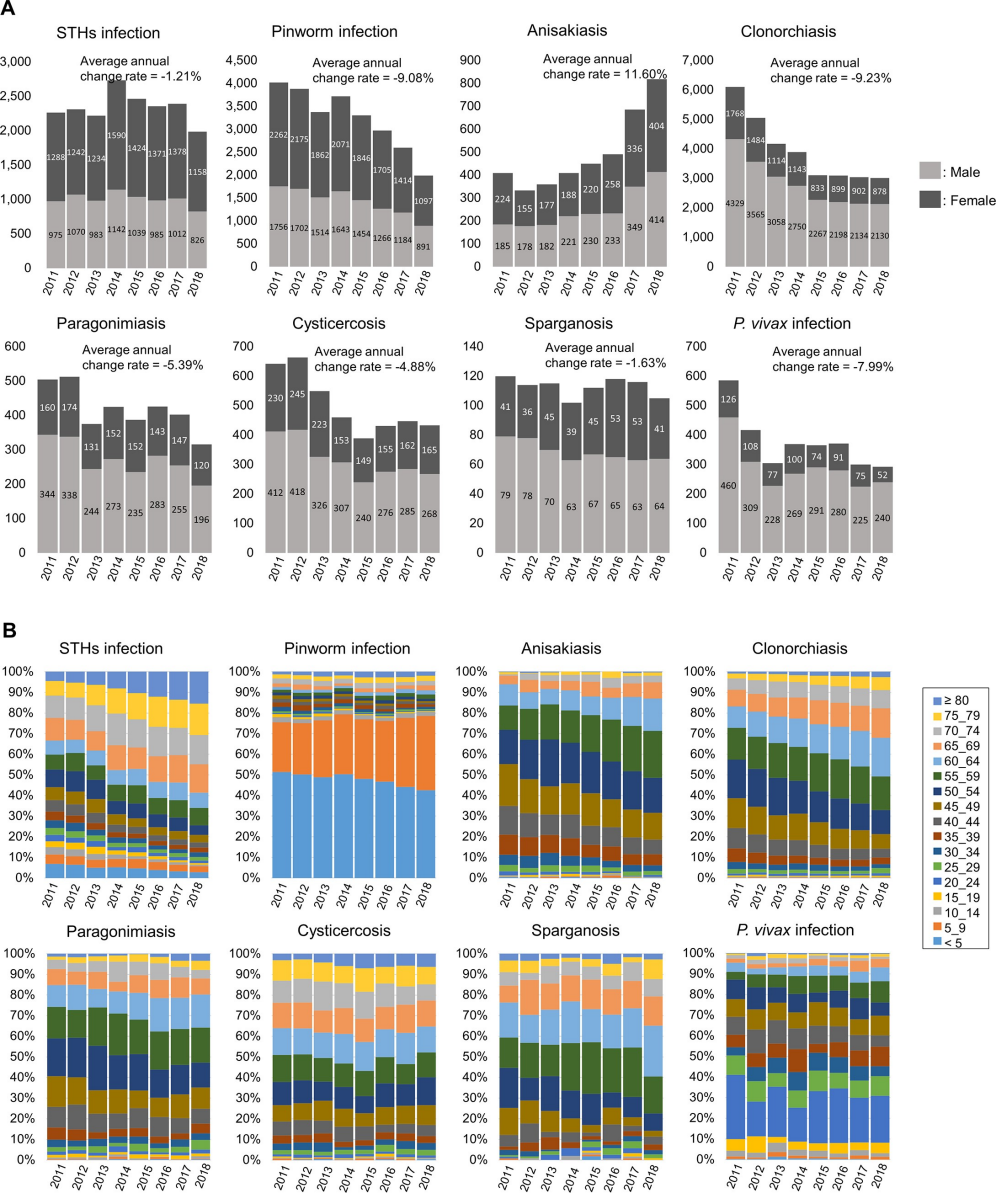
Number of patients infected with endemic parasites **(A)** and their age composition **(B)** in South Korea from 2011 to 2018.

Fig 1 shows the number of patients infected with various parasites in South Korea from 2011 to 2018.

The number of patients with STH infections remained around 2,300 for the 8-year period (Fig 1A). The number of patients infected with pinworm declined from 4,018 in 2011 to 1,988 in 2018 with an average annual change rate as -9.08%. The number of those with anisakiasis, however, increased from 409 in 2011 to 818 in 2018, increasing by 11.60% annually on average. The number of clonorchiasis cases decreased from 6,097 in 2011 to 3,008 in 2018 (-9.22% annually), and the number of paragonimiasis cases decreased from 504 in 2011 to 316 in 2018(-5.39% annually). The number of cysticercosis cases decreased from 642 in 2011 to 433 in 2018. The number of patients with sparganosis remained constant, at over 100 patients per year. The number of patients infected with *P*. *vivax* declined from 586 in 2011 to 292 in 2018 and its average annual rate of change was -7.99%. The numbers of clonorchiasis, paragonimiasis, cysticercosis, and sparganosis infections contracted by eating the intermediate host animals were much higher in men. Patients with *P*. *vivax* infection were also more likely to be men, because many were soldiers who were near the Demilitarized Zone (DMZ). However, the infection rate of anisakiasis was almost the same between men and women.

Pinworm infections affected mostly children, with 80% of patients being under 10 years of age. The infection rate in children who were younger than 5 years of age declined slightly over time (Fig 1B). The majority of patients with *P*. *vivax* infections were 20 to 24 years old (20% to 30%), attributed to the high number of soldiers near the DMZ. Other parasitic infections were more common in elderly people. Especially in the case of STHs, anisakiasis, clonorchiasis, and paragonimiasis, the proportion of cases in the elderly population increased over the years.

**Fig 2:**
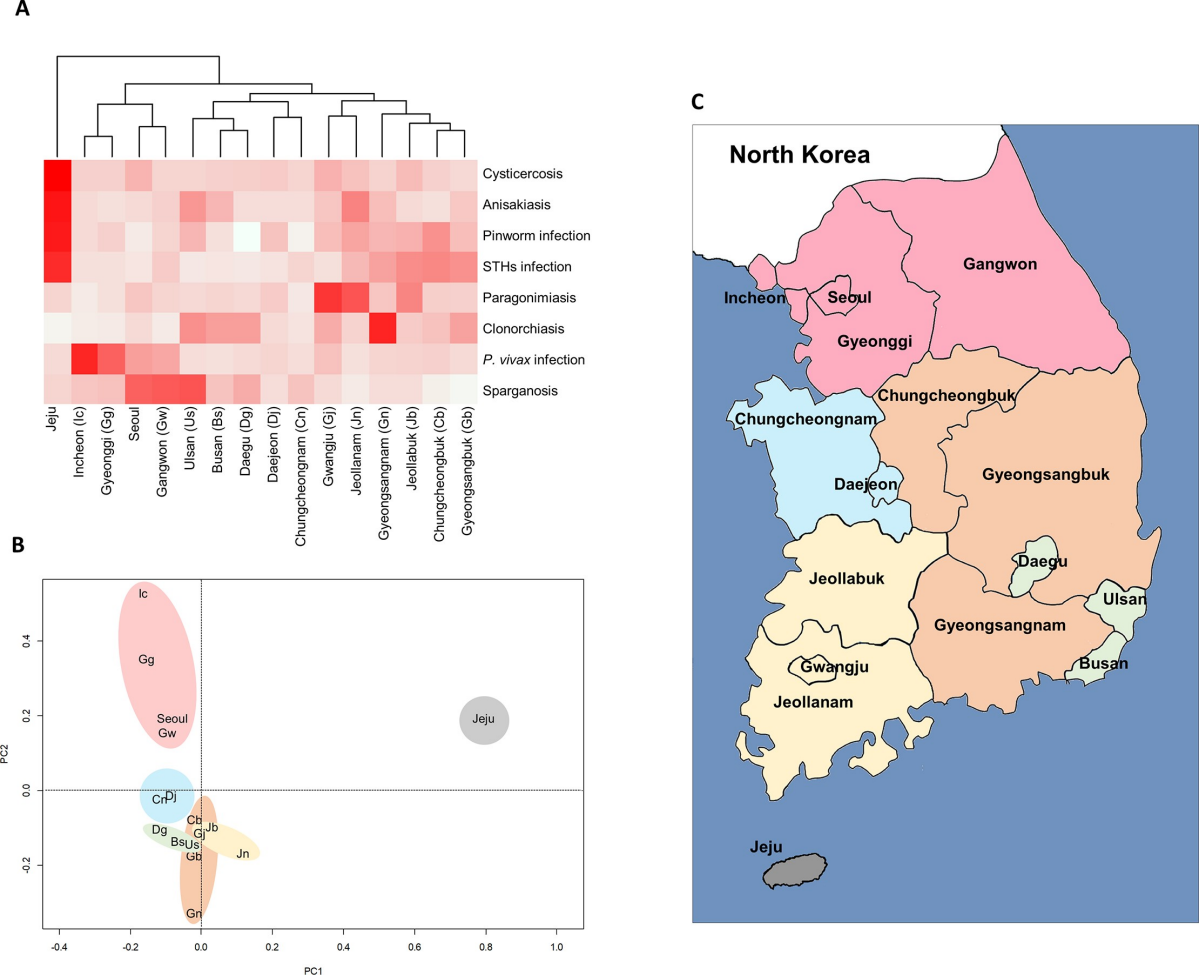
Claims frequency of endemic parasitic infections by province in South Korea. **(A)** Heatmap and dendrogram for the claims frequency of each parasite infection by province. Columns indicate the hierarchical clustering of the provinces that have a common pattern of claims frequency regarding parasite infection. **(B)** Principal component analysis (PCA) revealed claims frequency of parasitic infections by province. PCA showed that some provinces have similar parasitic infection patterns. **(C)** Map of South Korea representing patterns of endemic parasite infections analysed using the Heatmap (A) and PCA (B) data. Provinces with similar parasite infection patterns appear in the same color. The outline of the map of South Korea was manually drawn, and the map was colored using Microsoft Paint according to the relevant data.

The infection rate of endemic parasitic infections by province was analyzed using Heatmap and PCA (Fig 2).

Jeju province had the highest number of claims for cysticercosis, anisakiasis, pinworm infection, and STHs infections and showed a distinct parasitic frequency pattern according to Heatmap (Fig 2A) and PCA (Fig 2B). Incheon, Gyeonggi-do, Seoul, and Gangwon-do, close to North Korea, had higher frequency of claims for *P*. *vivax* infection than did other regions (Fig 2A), and were closely located according to PCA (Fig 2B), so these provinces were marked in pink on the map (Fig 2C). Gwangju, Jeollanam-do, and Jeollabuk-do had the highest number of claims for paragonimiasis. Clonorchiasis rates were highest in Gyeongsangnam-do and it was prevalent in southeast Korea, including Gyeongsangbuk-do, Ulsan, Busan, and Daegu (Fig 2A). STHs infection was prevalent in provinces that had a lot of farmland, such as Gyeongsangnam-do, Gyeongsangbuk-do, Jeollanam-do, Jeollabuk-do, and Chungcheongbuk-do. In Daejeon and Chungcheongnam-do, rates of all parasitic infections were low.

### Medical expenses among patients with parasitic infections

**Fig 3:**
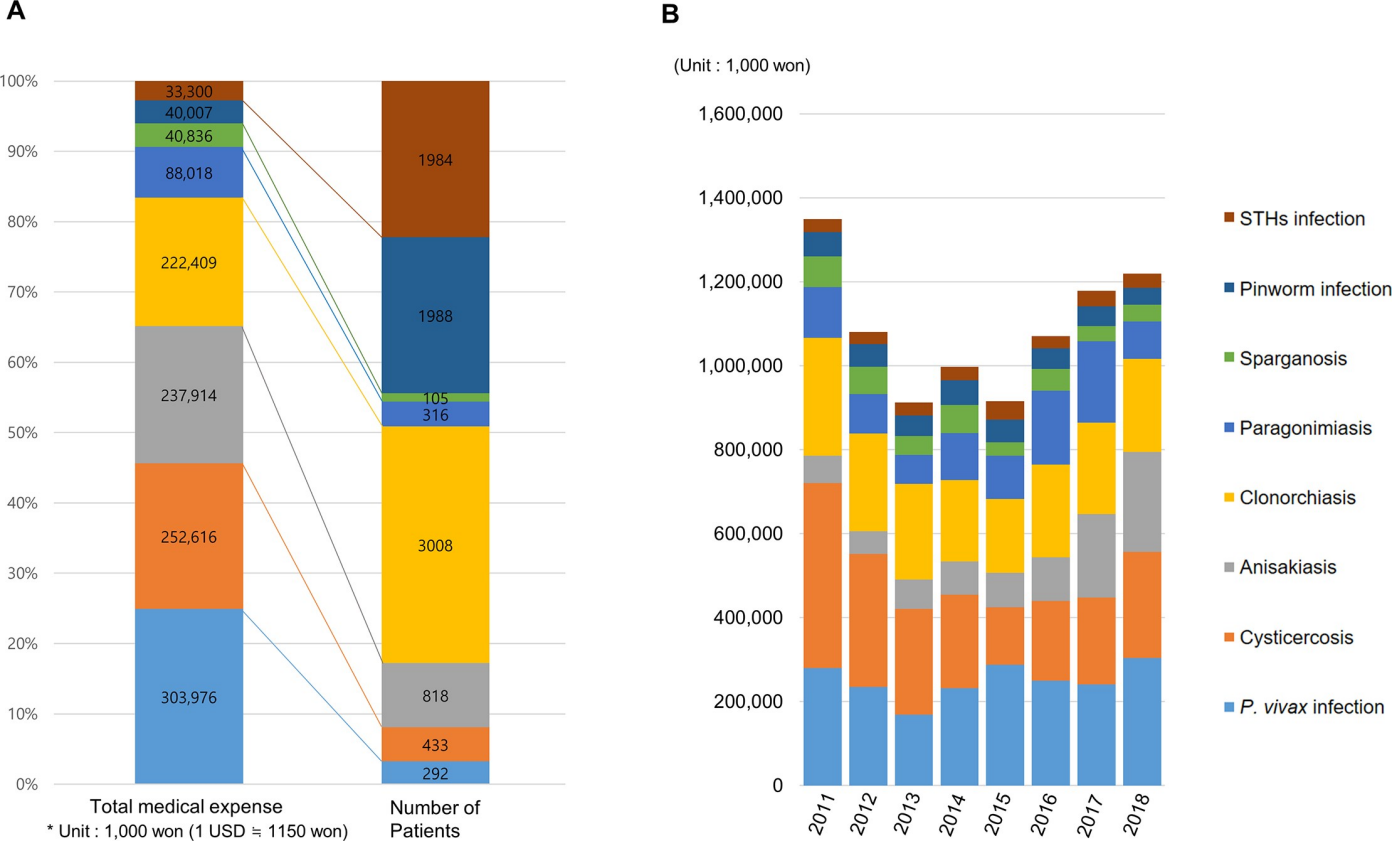
Total medical expense associated with endemic parasitic infections in South Korea. **(A)** Total medical expense and the number of patients in 2018. **(B)** Total medical expense from 2011 to 2018. The unit of money is 1,000 Korean won (1 US$ ≒ 1150 won).

Data on total medical expense and the number of patients (Fig 3A) showed that *P*. *vivax* infection, cysticercosis, and anisakiasis affected a small number of patients but caused relatively higher medical expenses. STHs infection, which is relatively easy to diagnose and treat, affected a large number of patients but caused only a small medical expense. In 2018, anisakiasis was the third most costly parasitic disease (237 million won), followed by clonorchiasis (222 million won), a traditional endemic disease.

In the time series trends for the total medical expense of endemic parasitic diseases from 2011 to 2018 (Fig 3B), the average sum of the medical expense for the eight endemic parasitic diseases was about 1.1 billion won per year (about 950,000 US$). The cost of cysticercosis and sparganosis declined during this time, while that of anisakiasis increased sharply. In 2011, the total medical expense for anisakiasis in South Korea was 65 million won, but it rose to 237 million won in 2018, increasing by 23.87% annually on average. Anisakiasis, together with malaria due to *P*. *vivax*, will likely be the most costly parasitic diseases in South Korea in the future.

### Trend of anisakiasis

The total number of anisakiasis patients was 818 in 2018, up from 409 in 2011 (Fig 1A). The infected patients were mainly middle-aged people (aged 40 to 59 years), composing 62.6% of patients in 2011 and 59.8% of patients in 2018 (Fig 1B). Due to the increasing trend of infection with age, the most infected age range in 2011 was 45–49 years (83 persons, 20.3%), and in 2018 it was 55–59 years (186 persons, 22.7%). Furthermore, anisakiasis was evaluated as a parasitic disease with one of the highest medical expenses in South Korea (Fig 3).

**Fig 4:**
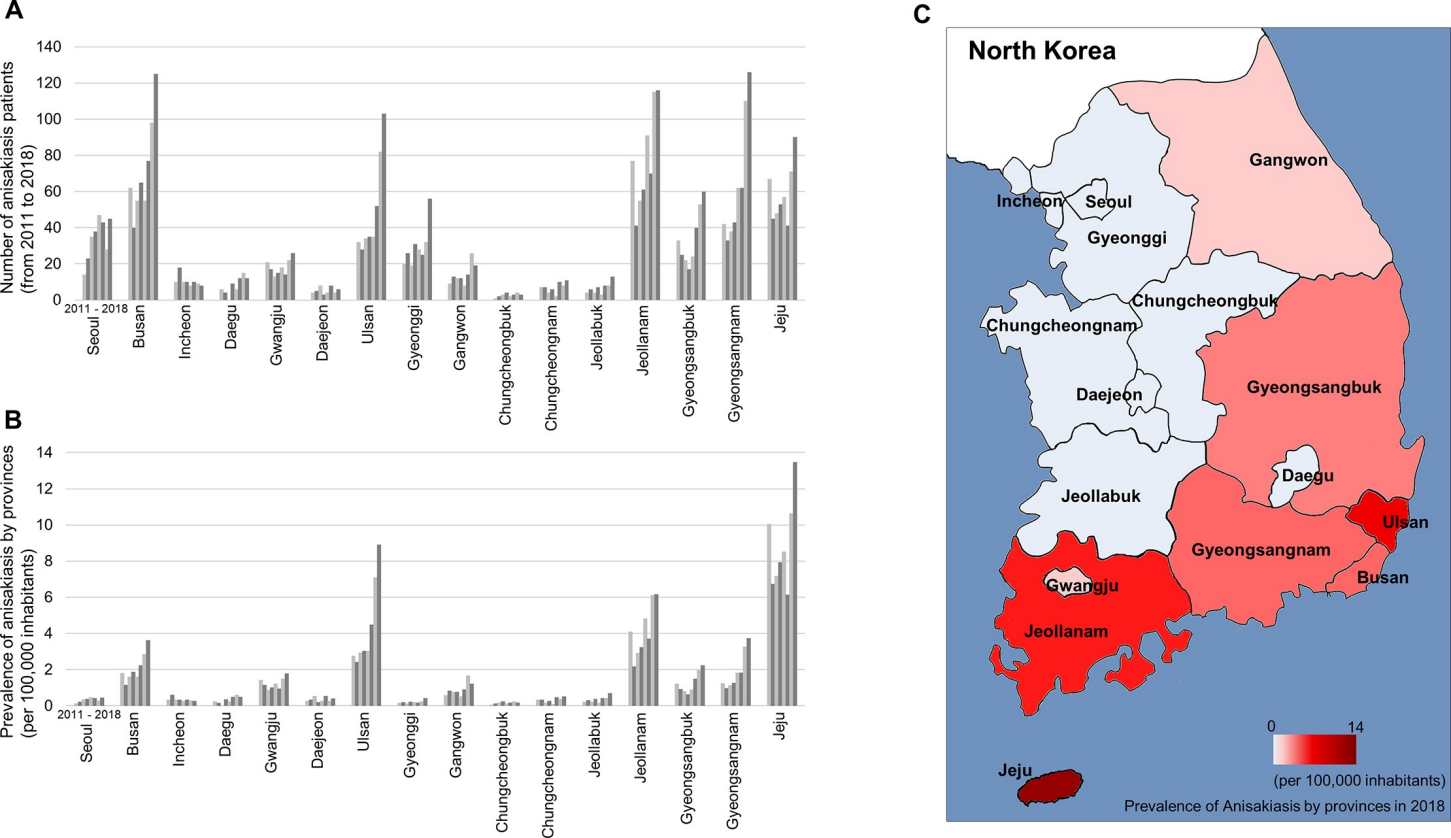
Trend of anisakiasis by province in South Korea. **(A)** The number of anisakiasis patients from 2011 to 2018. **(B)** Claims frequency for anisakiasis by province per 100,000 inhabitants from 2011 to 2018. **(C)** Map of South Korea representing the claims frequency for anisakiasis in 2018 by province. The outline of the map of South Korea was manually drawn, and the map was colored using Microsoft Paint according to the relevant data.

Fig 4 shows the trend of anisakiasis by province in South Korea from 2011 to 2018. Anisakiasis patients increased nationwide during this time (Fig 4A). In terms of claims frequency, Jeju province had the highest number of cases, followed by Ulsan and Jeollanam-do (Fig 4B). Anisakiasis had high frequency in southern Korea and was rare in western regions (Fig 4C). This phenomenon is thought to be influenced by the dietary habits of the inhabitants and the differences in fish species caught, the intermediate hosts of *Anisakis* spp.

### Medical expenses among patients with protozoan and ectoparasitic infections

**Fig 5:**
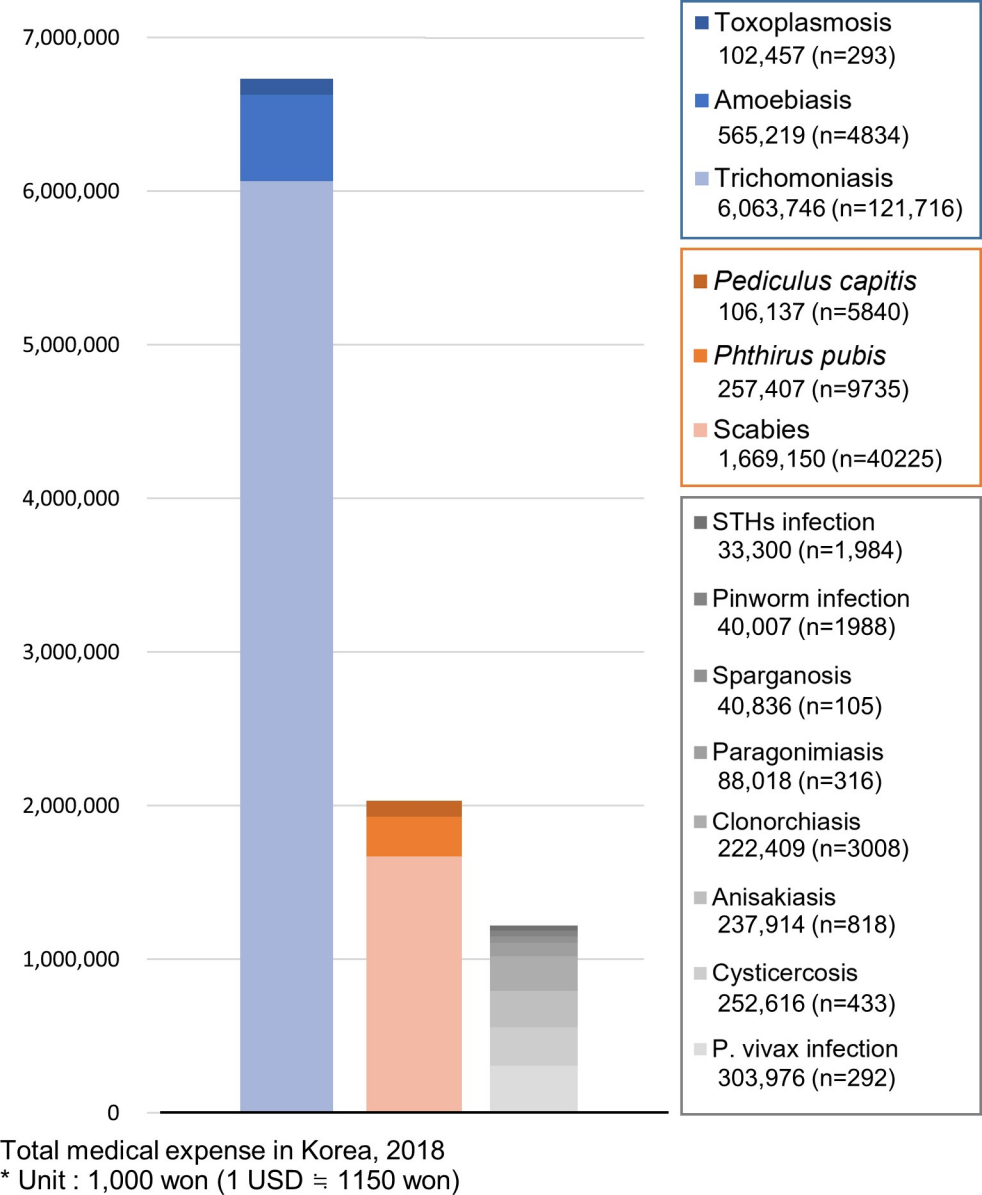
Total medical expense in South Korea due to parasitic diseases in 2018: globally distributed protozoan diseases (blue), ectoparasite infestations (orange), and endemic parasitic diseases (gray). The unit of money is 1,000 Korean won (1 US$ ≒ 1150 won). n = the number of patients.

Protozoan diseases with a worldwide distribution, such as toxoplasmosis, amoebiasis, and trichomoniasis, caused a total medical expense of 6,731 million won (≒ 5.85 million US$) in South Korea in 2018 (Fig 5). The largest portion of the expense was due to trichomoniasis, which cost approximately 6 thousand million won and affected 121,716 patients. The total medical expense of these protozoan diseases was 5.5 times the sum of those for endemic parasitic diseases (1,219 million won). Ectoparasite infestations including *P*. *capitis*, *P*. *pubis*, and scabies infestation caused a total medical expense of 2,032 million won. Scabies accounted for 82.1% of the sum of the total medical expenses for ectoparasite infestations. The sum of the total medical expenses for the ectoparasite infestations was 1.6 times that of the endemic parasitic diseases.

## Discussion

STH and pinworm infections declined during the 8-year study period. These patients accounted for 45% of the total endemic parasitic disease cases, but only 6% of the total medical expense. The highest claims frequency for STH and pinworm infections was in Jeju province. STHs infected more elderly people and were more prevalent in Gyeongsang-do and Jeolla-do provinces, which have a great deal of farmland. STHs such as *A*. *lumbricoides*, *T*. *trichiura*, and hookworm spread widely in South Korea in the 1950s and 1960s. Since that time, the management of these parasites has been supported by the government and Korea Association of Health Promotion. In 1969–1970, the rate of ascariasis in students was 55%, but it dramatically decreased to less than 10% in 1982 and 0.02% in 1995. *T*. *trichiura* and hookworm infections also showed similar patterns, declining from 65.4% and 10.7% in 1971 to 0.41% and 0% in 2004, respectively [1,12]. Positive rates of ascariasis, trichuriasis, and hookworm infection were 0.03%, 0.41%, and 0%, respectively in a national survey in 2012 [12]. In addition, 22 parasite infections were recently diagnosed by colonoscopy, including 16 *T*. *trichiura* and 6 *A*. *lumbricoides* infections, indicating that soil-mediated parasites are not completely eliminated in South Korea [13].

Foodborne parasitic diseases such as clonorchiasis are endemic parasitic diseases in South Korea. The present study determined that the number of clonorchiasis cases decreased from 6,097 in 2011 to 3,008 in 2018, but it was still the most prevalent helminthic parasite in South Korea. *C*. *sinensis* infections are acquired by humans who eat raw freshwater fish. It is prevalent in the Nakdonggang, Seomjingang, Youngsangang, and Geumgang rivers. The prevalence of clonorchiasis was 31.3% in regions near Nakdonggang river in 2006. According to the national survey, the prevalence was 4.6% in 1971, 2.6% in 1981, 2.9% in 2004, and 1.86% in 2012 [2,12,14,15]. The number of paragonimiasis cases, transmitted by ingesting raw freshwater lobsters and crabs, decreased from 504 in 2011 to 316 in 2018. In the 1960s, 2 million people were positive for this disease according to intradermal tests. The number of patients greatly decreased to fewer than 100 patients detected by ELISA per year from 1997 to 2007 [2,16]. Jeju province and seaside villages of Jeollanam-do, such as Gohung-gun and Wando-gun, were areas where paragonimus infection was prevalent.

The number of cysticercosis cases decreased from 642 in 2011 to 433 in 2018, with the highest frequency in Jeju province. Most of the cysticercosis cases were not newly diagnosed, but were previously diagnosed cases noted during follow-up treatment for conditions such as neurocysticercosis. Cysticercosis is caused by *Taenia solium* eggs and marked by tissue invasion. Neurocysticercosis can cause critical symptoms such as seizure and hydrocephalus. One medical center in South Korea reported 62 cases of cysticercosis from 1984 to 2005, 27 of which were cases of neurocysticercosis [17]. Another medical center reported 81 cases of neurocysticercosis from 1990 to 2016, and only eight cases were reported after 2010 [18]. A nationwide survey showed rates of infection with *Taenia* eggs of 1.8% in 1971, 0.27% in 1986, and 0.04% in 2012 [1,12] A survey of Jeju province alone showed an infection rate of 7.0% in 1988.

The number of sparganosis cases remained steady at over 100 patients per year. Sparganosis is contracted by eating raw snakes and frogs or drinking contaminated water. In South Korea, 119 cases of sparganosis were reported until 1989, and more cases were reported even after 2000 [17].

The number of patients infected with *P*. *vivax* declined from 586 in 2011 to 292 in 2018. The patients were more likely to be men in provinces near North Korea who were 20–24 years old. *P*. *vivax* malaria was eliminated in the late 1970s in South Korea [19,20]. However, since re-emerging indigenous malaria was reported in a soldier who worked in the DMZ in 1993, the incidence has increased steadily, reaching 4,142 cases in 2000 [21]. Among the 25,766 patients reported from 1993 to 2007, 14,709 were soldiers. Of the 21,419 patients reported from 1994 to 2005, 10,411 (48.6%) were in Gyeonggi-do province, 3,083 (14.4%) were in Gangwon-do province, and 2,710 (12.7%) were in Seoul [5,22].

Our analysis showed that anisakiasis was the parasitic disease that was most on the rise in South Korea. The total number of patients was 818 in 2018, up from 409 in 2011, and it was responsible for the largest total medical expense among parasitic diseases in South Korea in 2018, with costs expected to continue to grow in the future. *Anisakis* infects humans who eat raw seafood such as squid, mackerel, and conger. The detection rate of *Anisakis* has increased due to the recent use of endoscopy. The first recorded patient was infected after eating raw squid in 1971 in South Korea [23]. There were 107 cases reported in Jeju in 1995 and 141 cases in Gyeongsangnam-do in 2009 [24,25]. A review of the literature showed that 645 cases in South Korea have been reported in 64 articles [26]. The most favourable infection site for larvae was the stomach (82.4%) [26]. The common conger was the most probable source of human infections in South Korea (38.6%) [26]. *Anisakis pegreffii* is the predominant species causing human anisakiasis in South Korea [27].

Protozoan diseases such as trichomoniasis, amoebiasis, and toxoplasmosis caused a total medical expense of 6,731 million won (≒ 5.85 million US$) in 2018, 5.5 times that of endemic parasitic diseases. *Trichomonas vaginalis* is a parasite that infects the vaginas of women or urethras of men and is sexually transmitted. In South Korea, the infection rate was 35.8% in 1961 (according to outpatient data) and 3.4% in 1980 (according to a survey of soldiers [16,28]. *Entamoeba histolytica* is a waterborne infectious disease that has been prevalent in Korea in the past. This parasite can cause ulcers in the large intestine and severe diarrhea, as well as migrating to the liver where it can form an abscess. The prevalence was 4.3% in 1961 and 6.4% in 1971, according to stool analyses [16,29]. There were 159 amoebic liver abscesses reported in 1948 and 48 cases of amoebic enteritis in 1968 [16,29]. Patients with amoebic enteritis or liver abscesses have been rare in recent years. *Toxoplasma gondii*, contracted through the consumption of meat, can cause stillbirth and malformations in cases of congenital infection, and in adults it causes chorioretinitis, uveitis, and blindness. The positive rates of *Toxoplasma* infection were 5.8% in 1960, 4.3% in 1982, and 4.3% in 1996 [30–32]. Cases of ocular toxoplasmosis and congenital infection have been steadily reported in South Korea [33–36].

The sum of the total medical expenses due to *P*. *capitis*, *P*. *pubis*, and scabies infestations was 1.6 times that of the endemic parasitic diseases. *P*. *capitis* spreads easily among elementary school students. The prevalence of *P*. *capitis* infestation was 22.4% among 12,865 children in 1989. In 1995, it was 5.0% among 1,530 elementary school students. In 2003, 5.8% of 7,495 children had *P*. *capitis* [37,38]. *P*. *pubis* is a parasite that lives on genital hairs and is transmitted by sexual contact. Scabies cases rapidly decreased after the 1980s as hygiene improved in South Korea, but they have increased in recent years [39]. The recent increase of scabies is attributed to the inexperience of physicians and the increasing number of elderly patients admitted to nursing homes and eldercare hospitals [40].

The major strength of the present study was the utilization of a national claims database (HIRA data), which covers the entire South Korean population without selection bias. By using HIRA data, we were able to investigate important parasitic infections such as anisakiasis, which were not recognized previously by KCDC and medical communities.

However, the use of data from HIRA claims has limitations. Discrepancies have been reported between diagnoses in the claims database and actual diseases contracted by patients [6]. A previous study on medical discrepancies determined that, on average, 70% of diagnoses correspond to those in the patients’ medical records [6]. Furthermore, this approach might overlook asymptomatic infections; hence, results presented here should not be considered as actual prevalence because it was deduced from the claims data. Moreover, certain medical costs may not have been accounted for in the claims database.

In addition, the location where a beneficiary received healthcare services might differ from the location where the beneficiary lives. HIRA statistics are based on the location of the healthcare providers, not patients’ residences.

Although for these reasons HIRA data cannot capture the exact number of patients, they provide enough information to identify patients with these diseases in accordance with age, sex, and region, and shows temporal trends of disease incidence. Regarding the total medical expense, even considering misdiagnoses and overdiagnoses, HIRA data is best source of data that includes medical and social concerns about endemic parasitic disease. These endemic parasitic diseases cannot be ruled out during practical diagnosis, which causes an actual burden to the country in terms of total medical expenses even in the case of rare diseases. Therefore, the medical expenses related to the parasitic diseases investigated in the present study are valuable information for both public health and academic concerns.

In conclusion, the present study used HIRA claims data to show the trends for parasitic diseases in South Korea over an 8-year period. The analysis provides valuable information that can establish public health policy in Korea. These approaches using medical big-data can be applied to datasets in other countries to establish programs for infectious disease control, including control of parasitic diseases.

## Supporting information

S1 Table The number of patients in 2011–2018. (XLSX)

S2 Table The age composition among patients with parasitic infections. (XLSX)

S3 Table The parasitic infections by provinces. (XLSX)

S4 Table The medical expenses. (XLSX)
